# Survival Outcomes and Prognostic Analysis Following Greater Cytoreductive Radiotherapy in Patients With Metastatic Prostate Cancer

**DOI:** 10.3389/fonc.2020.549220

**Published:** 2020-09-30

**Authors:** Zitong Zhang, Min Wei, Lixin Mai, Yonghong Li, Jianhua Wu, Hong Huang, Sijuan Huang, Maosheng Lin, Xiaobo Jiang, Fangjian Zhou, Mengzhong Liu, Yang Liu, Liru He

**Affiliations:** ^1^State Key Laboratory of Oncology in South China, Department of Radiation Oncology, Sun Yat-sen University Cancer Center, Collaborative Innovation Center for Cancer Medicine, Guangzhou, China; ^2^Department of Oncology, The Seventh Affiliated Hospital, Sun Yat-sen University, Shenzhen, China; ^3^State Key Laboratory of Oncology in South China, Department of Urology, Sun Yat-sen University Cancer Center, Collaborative Innovation Center for Cancer Medicine, Guangzhou, China

**Keywords:** prostate cancer, metastasis, radiotherapy, cytoreduction, prognosis

## Abstract

**Purpose:** To assess the survival outcomes of patients with metastatic prostate cancer (mPCa) who undergo greater cytoreductive radiotherapy in a real-world clinical practice and determine their prognostic factors.

**Methods:** We performed a retrospective study of 160 patients with mPCa who underwent cytoreductive radiotherapy between 2009 and 2018 at a single institution. The degree of the cytoreductive burden was calculated for each patient. Overall survival (OS) was calculated from the date of detection of metastases. Variables associated with prostate-specific antigen (PSA) response and OS were evaluated via univariate and multivariate analyses.

**Results:** The median follow-up period was 47.2 months. The median OS was 42.3 months with a 5-year OS rate of 37.9%. The PSA levels of 90 patients (56.7%) decline by > 50% after radiotherapy. The 5-year OS rates of patients who underwent total, major, and minor cytoreductive radiotherapy were 53.4, 38.2, 17.6%, respectively; the corresponding median OS intervals were 62.5, 41.0, and 24.4 months, respectively (*P* < 0.001). A greater extent of cytoreduction (*P* < 0.05), lower PSA at radiotherapy initiation [hazard ratio 0.51, 95% confidence interval [CI] 0.33–0.78; *P* = 0.002] and better PSA response [hazard ratio 0.47, 95% CI 0.30–0.72; *P* < 0.001] were independent factors associated with superior OS. A high metastatic burden (as defined in the CHAARTED trial) was the only independent predictor of a poorer PSA response (odds ratio 0.36, 95% CI 0.19–0.69; *P* = 0.002). Grade 2 late gastrointestinal and genitourinary toxicities were observed in 3 and 2 patients, respectively, and only 1 patient had grade 3 late gastrointestinal toxicity.

**Conclusion:** Cytoreductive radiotherapy is effective and safe in select patients with mPCa. Greater cytoreduction, together with lower PSA at radiotherapy initiation and improved PSA response are favorable prognostic factors. Further studies are needed to confirm our findings.

## Introduction

Systemic therapy alone is currently the standard management approach for metastatic prostate cancer ([m]PCa). However, emerging evidence suggests that cytoreductive prostate radiotherapy, also known as primary-directed radiotherapy (PDRT), can delay disease progression in patients who present with osseous metastases and can even lead to improved overall survival (OS) in those with low metastatic burdens (1, 2). On the other hand, some prospective phase II studies have also shown the benefit of metastasis-directed radiotherapy (MDRT) in delaying disease progression for patients with oligometastatic recurrence (3, 4). More recently, stereotactic ablative radiotherapy (SABR) was reported to be associated with an improved OS in patients with oligometastatic cancers, including oligometastatic PCa (5). These encouraging results indicate that cytoreductive radiotherapy may be more useful than previously appreciated for the treatment of mPCa.

Nowadays, multisite radiation is safe and feasible using state-of-the-art radiotherapy techniques. Clinically, radiotherapy is often administered to both the primary and metastatic lesions of patients with mPCa; the treatment is not only for palliation but also for cytoreduction and is even administered with a curative intent in some cases. In several other metastatic and recurrent cancers, the percentage of treated tumor burdens (TBs) was found to be associated with improved outcome (6–8); however, the association between the extent of cytoreduction and any clinical benefit in patients with mPCa has not been identified, and little is known about the effect of greater cytoreductive radiotherapy on the outcomes of patients with mPCa except for a recent respective study with a small sample size which reported that combined PDRT and MDRT delayed disease progression than PDRT alone in the oligometastatic setting (9). Our present study aimed at assessing the survival outcomes and identifying prognostic factors when applying cytoreductive radiotherapy to patients with mPCa in a real-world clinical setting.

## Patients and Methods

### Patient Selection and Pre-treatment Evaluation

We reviewed the medical records of 639 patients with PCa who received radiotherapy between August 2009 and March 2018 at Sun Yat-sen University Cancer Center. Eligible patients were diagnosed with mPCa and received radiotherapy for at least 1 tumor lesion. Patients with other uncontrolled malignancies or those who underwent surgery after the detection of metastasis, were excluded. Patients were also excluded if they were followed up for < 3 months or had incomplete medical information. Ultimately, 160 patients were included for this study.

All patients had their diseases staged (or restaged) according to the 8th edition of the American Joint Committee on Cancer staging manual for PCa upon first detection of metastasis. The Gleason score was assessed (or reassessed) by the pathologists at our hospital according to the International Society of Urological Pathology grading criteria for PCa (10). Castration-resistant prostate cancer (CRPC) was diagnosed according to the definition used in the European Association of Urology guidelines on prostate cancer (11). Primary and metastatic tumor lesions were detected by ^18^F-fluorodeoxyglucose positron-emission tomography/computed tomography (PET/CT), ^68^Ga-labeled prostate-specific membrane antigen PET/CT, or Tc99m bone scintigraphy combined with magnetic resonance imaging (MRI) and/or CT.

The numbers of osseous metastases detected on bone scintigraphy were divided into 2 categories: <5 and ≥ 5 lesions based on the subgroup analysis findings of the HORRAD trial (1). Metastatic burden was classified according to the definitions used in the STAMPEDE and CHAARTED trials: high metastatic burden was defined as 4 or more osseous metastases with 1 or more present outside the vertebral bodies or pelvis, or visceral metastases, or both; all other assessable patients were considered to have a low metastatic burden (12). Risk classification was performed according to the definition used in the LATITUDE trial: high-risk was defined as the presence of at least 2 of 3 prognostic factors (Gleason score ≥ 8, 3 or more lesions identified by bone scanning, or measurable visceral metastases in locations other than the lymph nodes); all other assessable patients were categorized as being high-risk (13). This study was approved by the ethics committee of Sun Yat-sen University Cancer Center (B2020-074).

### Treatment Approaches

All patients received lifelong androgen deprivation therapy (either via orchidectomy or gonadotrophin-releasing hormone agonist treatment). For those with hormone sensitive prostate cancer (HSPC), androgen deprivation was applied with or without bicalutamide. Docetaxel was applied in addition to hormone therapy as one of the treatment choices for patients with high metastatic burdens. For those with metastatic CRPC, abiraterone acetate, docetaxel, or estramustine phosphate was used as a second-line treatment.

Cytoreductive radiotherapy was indicated for curative intent, local consolidation, or symptom palliation on the basis of first or second systematic therapy. All patients underwent 3 mm slice thickness contrast-enhanced CT and/or MRI simulation scanning with site-specific immobilization. Clinical target volumes were contoured according to the recommendations of the Radiation Therapy Oncology Group (RTOG). For PDRT, the clinical target volume (CTV) included the prostate with or without seminal vesicles. The CTV was expanded by 5 mm (3 mm posteriorly) to delineate the planning target volume. The pelvic lymph nodes were not included except for those positive for metastases. The prescribed dose was 60–67.5 Gy in 25 fractions for the prostate and 45–60 Gy in 25 fractions for any included pelvic lymph nodes; the overall treatment time was 5 weeks. SABR was generally used for MDRT. The CTV was equivalent to the gross tumor volume, and the planning target volume was defined as the CTV plus a variable margin (up to 5 mm). The prescribed dose ranged from 18 to 35 Gy in 1 to 5 fractions depending on the target size and location. Biologically effective dose (BED) was calculated using linear-quadratic model with α/β ratio of 2Gy (14). Volumetric intensity-modulated arc therapy was used for planning, and normal tissue dose constraints according to the RTOG guidelines were used. Cone beam CT was mandatory for daily image-guided radiation therapy.

The TB was defined as the sum of the longest unidimensional diameters of the target lesions according to Response Evaluation and Criteria in Solid Tumors (RECIST) version 1.1. Furthermore, the degree of the cytoreductive burden was calculated using the formula TB_cytoreductive radiotherapy_/TB_whole body_ and was divided into 3 categories: total cytoreduction (100%), major cytoreduction (>50%), and minor cytoreduction (≤50%) (15).

### Outcome Evaluations

The duration of follow-up was calculated from the time of diagnosis of mPCa. Generally, patients were regularly followed every 3 months for the first 2 years and every 6 months thereafter, although symptom-monitoring was performed on a weekly basis during radiotherapy. Prostate-specific antigen (PSA) levels were measured at every regular follow-up visit; imaging was prescribed when necessary at the clinician's discretion.

PSA response after radiotherapy was evaluated using the Prostate Cancer Working Group 2 criteria and was divided into 2 categories: a > 50% decline from baseline vs. a ≤50% decline (including PSA progression) (16). PSA progression was defined according to Prostate Cancer Working Group 2 criteria as the date that a 25% or greater increase and an absolute increase of 2 ng/mL or more from the nadir is documented. The time to PSA progression was measured from the initiation of radiotherapy until PSA progression. OS was defined as the interval between the diagnosis of metastases until death from any cause. Radiographic progression-free survival was measured from the initiation of radiotherapy until radiographic progression or death from mPCa. Radiographic progression was defined as the first evidence of existing tumor progression and/or the detection of a new metastatic lesion according to the RECIST version 1.1. Adverse events during and after radiotherapy were evaluated and graded according to Common Terminology Criteria for Adverse Events Version 4.0.

### Statistical Analysis

Time-dependent areas under the curve (AUC) derived from receiver operating characteristic (ROC) analyses were generated to compare the capacity of different TB classifications to predict the prognosis. The optimal PSA cut-off value at radiotherapy initiation was determined by the point on its ROC curve that represented the maximum Youden index. Survival rates were estimated using the Kaplan-Meier method and compared using the log-rank test. Categorical data were compared using the chi-square test. Cox regression using a proportional hazards model as well as logistic regression models were used for univariate and multivariate analyses. *P* < 0.05 were considered significant. SPSS version 22.0 (IBM Corp., Armonk, NY) and the R statistical software (version 3.5.0) were used for all the statistical analyses.

## Results

### Patient and Treatment Characteristics

Table 1 shows the characteristics of the 160 patients. The median age at initial mPCa diagnosis was 68 years. The median and the optimal PSA cut-off values at radiotherapy initiation were 10.13 and 16.5 ng/mL, respectively. Of the 160 patients, 108 (67.5%) were T3–4, 77 (48.1%) were N1, and 137 (85.6%) were M1b. One hundred and five patients (66.6%) had a Gleason score of 8–10, and, 108 (67.5%) had CRPC. Sixty-three (39.4%), 75 (46.9%), and 91 (56.9%) of the patients had < 5 osseous metastases, a low metastatic burden, and a low-risk classification, respectively. Approximately 3-quarters of the patients (116, 72.5%) underwent both PDRT and MDRT. The majority (127, 79.4%) received extend cytoreductive radiotherapy (i.e., total or major), and more than half of the patients (90, 56.7%) had a PSA decline > 50% after radiotherapy. The median BED of the radiotherapy regimen dose prescribed to prostate and bone metastases was 149.5Gy (range 124.0–158.63Gy) and 100Gy (range 87.5–180.0Gy), respectively.

**Table 1:** Patient characteristics (*N* = 160).

**Parameter**	**Patients (%)**
Age, years	
Median (range)	68 (47–85)
T stage	
T2	52 (32.5)
T3	31 (19.4)
T4	77 (48.1)
N stage	
N1	77 (48.1)
N0	83 (51.9)
M stage	
M1a	14 (8.8)
M1b	137 (85.6)
M1c	9 (5.6)
WHO performance status	
0	90 (56.3)
1–2	70 (43.7)
Gleason sum score	
6–7	55 (33.4)
8–10	105 (66.6)
Hormone sensitivity	
HSPC	52 (32.5)
CRPC	108 (67.5)
Number of osseous metastases	
<5	63 (39.4)
≥5	97 (60.6)
Metastatic burden (CHAARTED)	
Low	75 (46.9)
High	85 (53.1)
Risk classification (LATITUDE)	
Low-risk	86 (53.8)
High-risk	74 (46.2)
PSA at radiotherapy initiation	
>16.5 ng/ml^*^	62 (38.7)
≤ 16.5 ng/ml	98 (61.3)
Radiotherapy site	
PDRT	9 (5.6)
PDRT + MDRT	116 (72.5)
MDRT (primary tumor previous controlled)	17 (10.6)
MDRT (primary tumor previous uncontrolled)	18 (11.3)
Cytoreductive extent	
Minor cytoreduction	33 (20.6)
Major cytoreduction	80 (50.0)
Total cytoreduction	47 (29.4)
PSA response after radiotherapy	
Decline ≤ 50%	70 (43.3)
Decline > 50%	90 (56.7)
Medications at radiotherapy	
ADT ± Bicalutamide	52 (32.5)
ADT + Abiraterone acetate	20 (12.5)
ADT + Docetaxel	23 (14.4)
ADT + Estramustine phosphate	65 (40.6)

HSPC, hormone sensitive prostate cancer; CRPC, Castration-resistant prostate cancer; PSA, Prostate-specific antigen; PDRT, Primary-directed radiotherapy; MDRT, Metastasis-directed radiotherapy; ADT, androgen deprivation therapy;

* *the optimal cutoff value of PSA at radiotherapy initiation defined by ROC curves*.

### Survival Outcomes and Univariate Analysis

**Figure 1:**
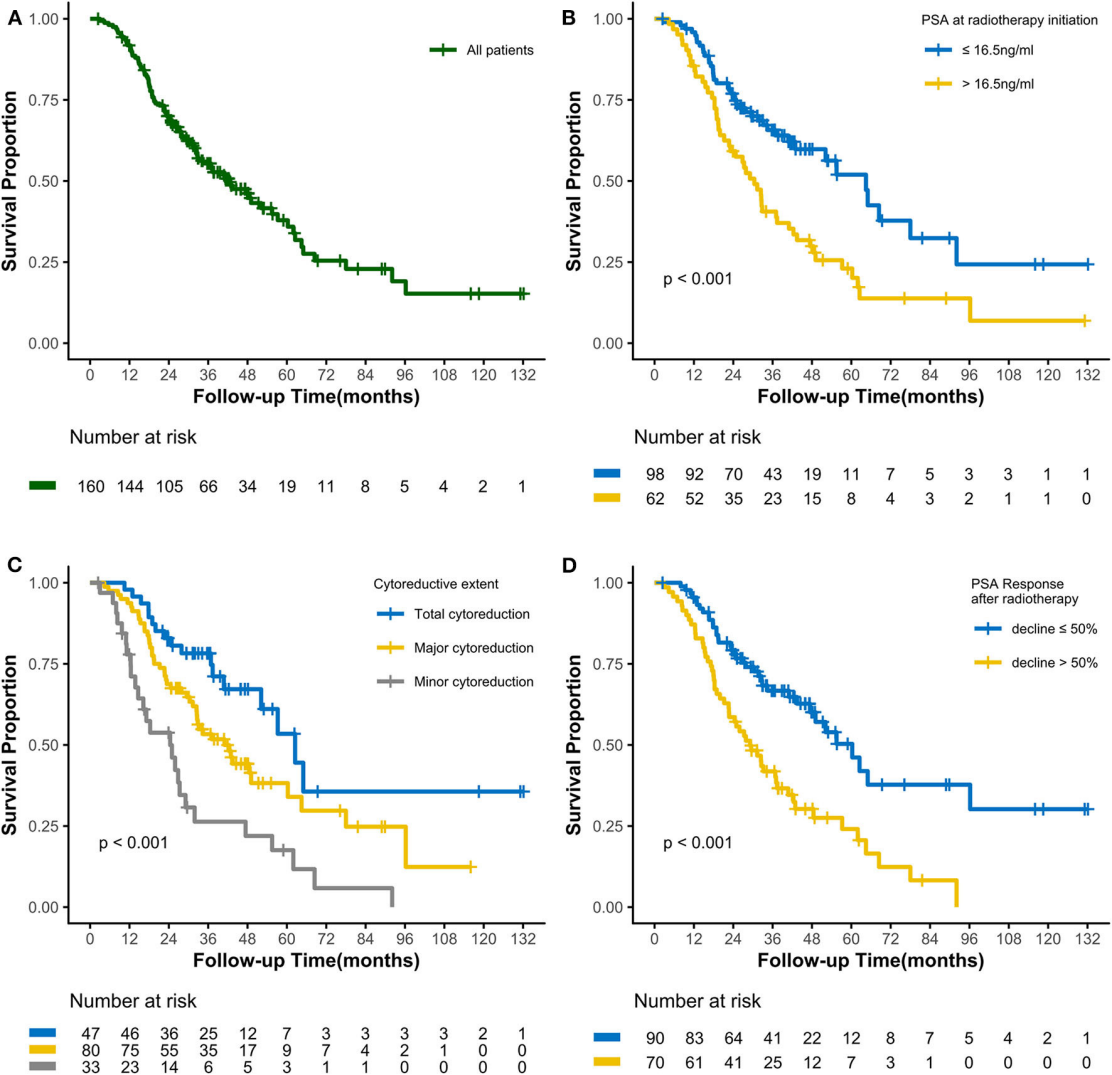
Kaplan-Meier curves calculating overall survival **(A)** of all patients, **(B)** by PSA at radiotherapy initiation, **(C)** by cytoreductive extent, and **(D)** by PSA Response after radiotherapy.

**Figure 2:**
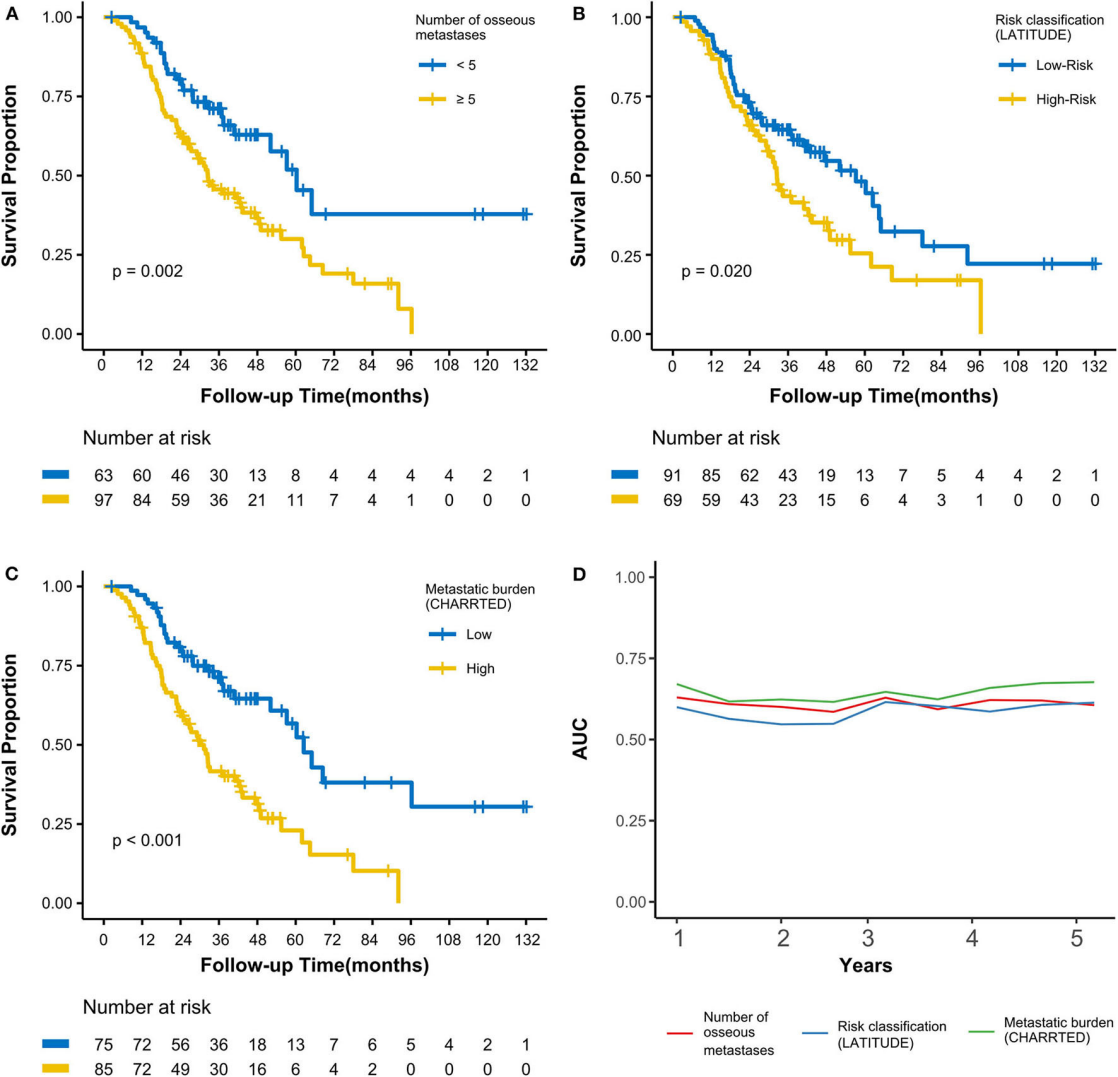
Kaplan-Meier curves calculating overall survival by **(A)** number of osseous metastases, **(B)** by risk classification (LATITUDE), **(C)** by metastatic burden (CHAARTED). **(D)** Time-dependent AUC curves based on three classifications for the first 5 years. AUC, area under the curve.

As of the last follow-up date of January 20th, 2020, 90 patients (56.3%) had died; the median follow-up period was 47.2 months. The median OS was 42.3 (3.0–127.7) months, with 3- and 5-year OS rates of 55.4 and 37.9%, respectively (Figure 1A). After radiotherapy, the median time to PSA progression was 7.1 months and the median radiographic progression-free survival was 13.4 months. Patients receiving total cytoreductive radiotherapy had a markedly longer median OS (62.5 months) than did those who underwent major (41.0 months) and minor (24.4 months) cytoreductive radiotherapy (*P* < 0.001); the corresponding 5-year OS rates were 53.4, 38.2, 17.6%, respectively (Figure 1C). Compared with those who had high PSA at radiotherapy initiation (>16.5 ng/mL), patients with low initial PSA had a longer OS on univariate analysis [hazard ratio [HR] 0.48, 95% confidence interval [CI] 0.32–0.72; *P* < 0.001, Figure 1B]. Patients with a PSA decline of > 50% had a significantly longer median OS than did those whose PSA levels declined by ≤50% after radiotherapy (HR 0.42, 95% CI 0.27–0.64; *P* < 0.001, Figure 1D). Patients with HSPC had a significantly longer median OS than did those with CRPC (HR 0.47, 95% CI 0.28–0.80; *P* = 0.006). Furthermore, univariate analyses demonstrated that the following parameters significantly impacted OS in the entire cohort: number of osseous metastases, risk classification (as defined in the LATITUDE trial), and metastatic burden (as defined in the CHAARTED trial) (all *P* < 0.05, Table 2, Figures 2A–C). Factors which impacted OS significantly in the entire cohort also had the same tendency in the cohorts of HSPC and CRPC. Besides, no significant difference of survival was observed between the high BED group and the low BED group.

**Table 2:** Univariate analysis for overall survival.

**Variables**	**ALL (*****N*** **= 160)**		**HSPC (*****N*** **= 52)**		**CRPC(*****N*** **= 108)**	
	**HR (95% CI)**	***p*-Value**	**HR (95% CI)**	***p*-Value**	**HR (95% CI)**	***p*-Value**
Gleason sum score		0.248		0.454		0.284
6–7	1		1		1	
8–10	1.30 (0.83–2.02)		1.50 (0.52–4.29)		1.31 (0.80–2.13)	
Age		0.559		0.585		0.843
≤ 68	1		1		1	
>68	1.14 (0.74–1.74)		1.32 (0.49–3.57)		1.05 (0.65–1.68)	
WHO performance status		**0.003**		0.196		**0.037**
0	1		1		1	
1, 2	1.88 (1.24–2.86)		1.90 (0.72–4.99)		1.65 (1.03–2.63)	
Cytoreductive extent		**<0.001**		**0.007**		**0.005**
Minor cytoreduction	1	Ref	1	Ref	1	Ref
Major cytoreduction	0.46 (0.29–0.75)	**0.002**	0.18 (0.04–0.70)	**0.014**	0.55 (0.33–0.93)	**0.025**
Total cytoreduction	0.25 (0.14–0.46)	**<0.001**	0.18 (0.06–0.65)	**0.009**	0.29 (0.13–0.63)	**0.002**
Number of osseous metastases		**0.002**		0.127		0.063
<5	1		1		1	
≥5	2.13 (1.33–3.42)		2.21 (0.80–6.11)		1.72 (0.97–3.04)	
Metastatic burden (CHAARTED)		**<0.001**		**0.034**		**0.005**
Low	1		1		1	
High	2.53 (1.62–3.96)		3.01 (1.09–8.35)		2.10 (1.25–3.53)	
Risk classification (LATITUDE)		**0.020**		0.062		0.335
Low-risk	1		1		1	
High-risk	1.64 (1.08–2.49)		2.73 (0.95–7.83)		1.26 (0.79–1.99)	
Hormone sensitivity		**0.006**				
CRPC	1					
HSPC	0.47 (0.28–0.80)					
PSA at radiotherapy initiation		**<0.001**		**0.043**		0.059
>16.5 ng/ml^*^	1		1		1	
≤ 16.5 ng/ml	0.48 (0.32–0.72)		0.27 (0.07–0.96)		0.63 (0.39–1.02)	
PSA response		**<0.001**		0.052		**0.002**
Decline ≤ 50%	1		1		1	
Decline > 50%	0.42 (0.27–0.64)		0.36 (0.13–1.01)		0.48 (0.30–0.77)	

*HSPC, hormone sensitive prostate cancer; CRPC, Castration-resistant prostate cancer; PSA, Prostate-specific antigen*.

* *The optimal cutoff value of PSA at radiotherapy initiation defined by ROC curves. Bold values represent the p-values that are statistically significance*.

### Multivariate Analysis of OS

To avoid multicollinearity among correlative parameters, ROC analysis was used to construct time-dependent AUC curves to assess the effect of 3 variables (number of osseous metastases, risk classification, and metastatic burden) on OS (Figure 2D). The mean AUCs from the first to the fifth years when assessing the number of osseous metastases, risk classification (LATITUDE), and metastatic burden (CHAARTED) were 0.61, 0.58, and 0.65, respectively. This showed that metastatic burden (CHAARTED), which had the highest AUC, was the most optimal prognostic predictor among the 3 parameters.

Thus, metastatic burden (CHAARTED) together with PSA at radiotherapy initiation, hormone sensitivity, cytoreductive extent, and PSA response were subjected to multivariate analysis. A greater extent of cytoreduction (*P* < 0.05), lower PSA at radiotherapy initiation [HR 0.51, 95% confidence interval [CI] 0.33–0.78; *P* = 0.002] and better PSA response (HR 0.47, 95% CI 0.30–0.72; *P* < 0.001) were found to be independent predictors of superior OS in the entire cohort (Table 3). After stratification according to castration status, a greater extent of cytoreduction remained to be significant predictors of superior OS in both the HSPC and the CRPC subgroups.

**Table 3:** Multivariate analysis for overall survival.

**Variables**	**ALL (*****N*** **= 160)**		**HSPC (*****N*** **= 52)**		**CRPC(*****N*** **= 108)**	
	**HR (95% CI)**	***p*-Value**	**HR (95% CI)**	***p*-Value**	**HR (95% CI)**	***p*-Value**
WHO performance status		0.242		0.167		0.391
0						
1, 2						
Cytoreductive extent						
Minor cytoreduction	1	Ref	1	Ref	1	Ref
Major cytoreduction	0.56 (0.34–0.92)	**0.022**	0.18 (0.04–0.70)	**0.014**	0.61 (0.36–1.05)	0.075
Total cytoreduction	0.35 (0.19–0.66)	**0.001**	0.18 (0.05–0.65)	**0.009**	0.33 (0.15–0.73)	**0.006**
Metastatic burden (CHAARTED)		0.165		0.123		0.377
Low						
High						
Hormone sensitivity		0.754				
CRPC						
HSPC						
PSA at radiotherapy initiation		**0.002**		0.715		**0.011**
>16.5 ng/ml^*^	1				1	
≤ 16.5 ng/ml	0.51 (0.33–0.78)				0.52 (0.32–0.86)	
PSA response		**<0.001**		0.053		**0.003**
Decline ≤ 50%	1				1	
Decline > 50%	0.47 (0.30–0.72)				0.47 (0.28–0.78)	

HSPC, hormone sensitive prostate cancer; CRPC, Castration-resistant prostate cancer; PSA, Prostate-specific antigen;

* *The optimal cutoff value of PSA at radiotherapy initiation defined by ROC curves. Bold values represent the p-values that are statistically significance*.

### Predictors of PSA Response

Univariate analysis revealed that the World Health Organization performance status, number of osseous metastases, metastatic burden, and hormone sensitivity were significant predictors of PSA response (all *P* < 0.05, Table 4). Based on ROC curve analysis, metastatic burden was found to be a better predictor of PSA response than the number of osseous metastases as it had the larger AUC. On multivariate analysis, a high metastatic burden (CHAARTED) was found to be the only independent predictor of a poorer PSA response (odds ratio 0.36, 95% CI 0.19–0.69; *P* = 0.002, Table 4).

**Table 4:** Predictors estimating PSA response decline ≥ 50%.

**Variables**	**Univariate**		**Multivariate**	
	**analysis**		**analysis**	
	**OR (95% CI)**	***p*-Value**	**OR (95% CI)**	***p*-Value**
Gleason sum score		0.324		–
6–7	1			
8–10	1.39 (0.72–2.68)			
Age		0.855		–
≤ 68	1			
>68	0.94 (0.50–1.78)			
WHO performance status		**0.041**		0.177
0	1			
1, 2	0.52 (0.27–0.98)			
Cytoreductive extent		0.078		–
Minor cytoreduction				
Major cytoreduction				
Total cytoreduction				
Number of osseous metastases		**0.014**		–
<5	1			
≥5	0.44 (0.23–0.85)			
Metastatic burden (CHAARTED)		**0.002**		**0.002**
Low	1		1	
High	0.36 (0.19–0.69)		0.36 (0.19–0.69)	
Risk classification (LATITUDE)		0.365		–
Low-risk	1			
High-risk	0.75 (0.40–1.41)			
Hormone sensitivity		**0.008**		0.068
CRPC	1			
HSPC	2.56 (1.27–5.26)			

*HSPC, hormone sensitive prostate cancer; CRPC, Castration-resistant prostate cancer; PSA, Prostate-specific antigen. Bold values represent the p-values that are statistically significance*.

### Radiation-Related Toxicity

Acute toxicities occurred in 68 of the 160 patients (42.5%) overall. These were mainly acute gastrointestinal (GI) toxicity and genitourinary (GU) toxicities, although 5 patients presented with grade 1 myelosuppression. The incidences of grade 2 acute GI and GU toxicities were 7.2 and 4.0%, respectively, for patients treated with PDRT (*n* = 125). There were no grade ≥ 3 acute toxicities. Grade 2 late GI and GU toxicities were, respectively, observed in 3 and 2 patients, respectively, although only 1 had grade 3 late GI toxicity. Compression fractures were observed in 8 of the 151 patients (5.3%) treated with MDRT; all were asymptomatic and categorized as grade 1. No grade 4–5 toxicities were reported.

## Discussion

Owing to the polyclonal nature of the primary and metastatic lesions, the heterogenous responses to systematic therapy could still lead to tumor progression. Although effective drugs are available, cytoreductive radiotherapy plays an important role in the treatment of mPCa. Despite accumulating data from prospective studies that point to promising survival outcomes following PDRT or MDRT in select patients with mPCa (1–4), the evidence remains far from sufficient to formulate clinical practice guidelines. This study, which was based on real-world data from our institution, revealed superior treatment responses and survival outcomes in select patients with mPCa, and identified valuable prognostic factors for those who receive cytoreductive radiotherapy combined with systematic therapy.

It is well-accepted that cytoreductive radiotherapy might be most beneficial to patients with mPCa who have low metastatic burdens or oligorecurrent status (1–5). In the Stempede trial and the Horrad trial, radiotherapy to primary sites has been shown to improve survival in patients with limited metastasis (1, 2). In the STAMPEDE trail, the median OS in the PDRT group was significantly longer in patients with low TB (48 months). Delayed disease progression with PDRT was observed in all patients with osseous mPCa, even though visceral metastasis status was unknown at the time of randomization in the Horrad trial (1). An additional clinical benefit was previously observed after more aggressive cytoreductive therapy, independent of the number of metastatic sites, in some other human cancers (6–8). In our current study, the median OS of patients with HSPC and CRPC treated with cytoreductive radiotherapy was 52.1 and 32.8 months, respectively, indicating potential survival benefit brought by aggressive cytoreductive radiotherapy. The HORRAD and the STAMPEDE trial defined metastatic burden through conventional imaging, and patients in the low burden group might be polymetastatic under PET/CT scans (1, 2). Therefore, whether the potential benefit of cytoreductive radiotherapy is only limited to patients with low metastatic burdens remains unclear. In our study, not all patients receiving aggressive cytoreductive radiotherapy had a low TB, but survival benefit was observed in patients receiving aggressive local therapy. Thus, we cannot rule out the possibility that greater cytoreductive radiotherapy impacts clinical survival in patients mainly (but not exclusively) in those with low metastatic burdens or oligorecurrence.

Preclinical studies have demonstrated the existence of primary-metastasis and metastasis-metastasis communications between cancer cells and neighboring non-cancerous counterparts in a manner that could promote tumor progression (17). Apart from reducing the actual volume of the tumor, cytoreductive radiotherapy may also reduce the likelihood of lineage adaptation and thus improve patients' survival. On the other hand, multisite irradiation may release a wider range of tumor antigens and increase the possibility of activating the host immune system, thereby providing an additional benefit (18). In clinical studies, both PDRT and MDRT have demonstrated some degrees of clinical benefit in patients with oligometastatic PCa. A greater extent of cytoreductive radiotherapy combined with PDRT and MDRT may yield a better treatment outcome than would PDRT or MDRT alone. This notion is supported by a recently published retrospective study in which better CRPC-free survival rates were observed in patients with oligometastatic PCa treated with total cytoreductive radiotherapy than were observed in those receiving PDRT alone (9). However, the precise association between the extent of cytoreduction and any clinical benefit in patients with mPCa has not been identified. In our present study, we found that patients who received total cytoreductive radiotherapy had the best clinical outcomes, followed by those who received major cytoreductive radiotherapy; the extent of cytoreduction was found to be an independent predictor of patients' survival. Taken together, these data indicate that the degree of cytoreductive radiotherapy might impact the survival of patients with mPCa.

While cytoreductive radiotherapy appears to benefit select patients with mPCa, the most appropriate definition of low metastatic burden remains to be determined, and reliable prognostic factors also need to be verified. Since large clinical trials made no attempts to perform analyses using alternative definitions based on their published results, we classified our patients by metastatic burden using 3 different criteria based on the definitions used in the CHAARTED, LATITUDE, and HORRAD trials (1, 12, 13). We found that the CHAARTED definition, which took into account the sites and numbers of osseous metastases as well as the presence of visceral metastases, was the best predictor of prognosis among the 3, and was the only independent predictor of PSA response in our study. This may be expected given that the presence of visceral metastases is believed to be a poor prognostic factor (19), and metastatic burden cannot be judged merely based on the number of lesions. Nevertheless, the CHAARTED definition is not perfect since other important predictors of metastatic spread such as histological grade, tumor diameter, and local tumor stage may also play a role in determining TB. It would be more rational to assess the TB of the whole body instead of only assessing the metastatic burden when selecting patients for cytoreductive radiotherapy. Clearly, treatment-related factors including the treatment time and strategy would also impact patient survival. In our study, we found that lower PSA at radiotherapy initiation, greater cytoreductive extent, and a better PSA response were independent predictors of superior OS. Given that a low metastatic burden (as defined in the CHAARTED trial) was evaluated as the only independent predictor of PSA response, patients with a lower PSA at radiotherapy initiation and a low metastatic burden may be the best beneficiaries from cytoreductive radiotherapy. These data can help identify critical prognostic factors among patients with mPCa who are treated with cytoreductive radiotherapy combined with systematic therapy, although additional studies are still required for validation.

Our study had some inherent limitations. First, it was a retrospective, single-arm study with a relatively small sample size. The study might represent a select cohort, whom the care providers considered suitable for cytoreduction. Second, there was heterogeneity in our population since it was based on data from real-world clinical practice without differentiating between synchronous and non-synchronous metastases. Third, the TB was evaluated based on either conventional imaging or PET/CT; hence, the varying sensitivities of different imaging techniques should be taken into account. Furthermore, the study spanned a long period (2009–2018), during which systematic therapies changed and advanced. Additional studies are required to evaluate the role of cytoreductive radiotherapy in the treatment of mPCa.

## Conclusion

Our results showed that cytoreductive radiotherapy was effective and safe in select patients with mPCa. Greater cytoreduction, together with lower PSA at radiotherapy initiation and improved PSA response are favorable prognostic factors. Further studies are needed to confirm our findings.

## Data Availability Statement

The raw data supporting the conclusions of this article will be made available by the authors, without undue reservation.

## Ethics Statement

The studies involving human participants were reviewed and approved by ethics committee of Sun Yat-sen University Cancer Center. Written informed consent for participation was not required for this study in accordance with the national legislation and the institutional requirements.

## Author Contributions

Material preparation, data collection, and analysis were performed by ZZ, MW, LM, YLi, JW, HH, SH, MLin, and XJ. The original draft of the manuscript was written by ZZ and all authors commented on previous versions of the manuscript. Review and editing were performed by FZ, MLiu, YLiu, and LH. LH agree to be accountable for all aspects of the work in ensuring that questions related to the accuracy or integrity of any part of the work are appropriately investigated and resolved. All authors contributed to the study conception, design, read, and approved the final manuscript.

## Conflict of Interest

The authors declare that the research was conducted in the absence of any commercial or financial relationships that could be construed as a potential conflict of interest.
